# Patient-derived ovarian cancer xenografts re-growing after a cisplatinum treatment are less responsive to a second drug re-challenge: a new experimental setting to study response to therapy

**DOI:** 10.18632/oncotarget.7465

**Published:** 2016-02-17

**Authors:** Francesca Ricci, Maddalena Fratelli, Federica Guffanti, Luca Porcu, Filippo Spriano, Tiziana Dell’Anna, Robert Fruscio, Giovanna Damia

**Affiliations:** ^1^ Department of Oncology, Laboratory of Molecular Pharmacology, IRCCS-Istituto di Ricerche Farmacologiche Mario Negri, Milan, Italy; ^2^ Department of Biochemistry, IRCCS-Istituto di Ricerche Farmacologiche Mario Negri, Milan, Italy; ^3^ Department of Oncology, Laboratory of Methodology for Biomedical Research, IRCCS-Istituto di Ricerche Farmacologiche Mario Negri, Milan, Italy; ^4^ Obstetrics and Gynecology Clinic, San Gerardo Hospital, Monza, Italy

**Keywords:** patient-derived xenografts, ovarian carcinoma, cisplatinum resistance, epithelial-mesenchymal transition, cancer stem cells

## Abstract

Even if ovarian cancer patients are very responsive to a cisplatinum-based therapy, most will relapse with a resistant disease. New experimental animal models are needed to explore the mechanisms of resistance, to better tailor treatment and improve patient prognosis. To address these aims, seven patient-derived high-grade serous/endometrioid ovarian cancer xenografts were characterized for the antitumor response after one and two cycles of cisplatinum and classified as Very Responsive, Responsive, and Low Responsive to drug treatment. Xenografts re-growing after the first drug cycle were much less responsive to the second one. The expression of epithelial-mesenchymal transition (EMT) and cancer stem cells (CSCs) genes was investigated in cisplatinum-treated and not-treated tumors. We found that different EMT (*TCF3*, *CAMK2N1*, *EGFR*, and *IGFBP4*) and CSCs (*SMO*, *DLL1*, *STAT3*, and *ITGA6*) genes were expressed at higher levels in Low Responsive than in Responsive and Very Responsive xenografts. The expression of STAT3 was found to be associated with lower survival (HR = 13.7; *p* = 0.013) in the TCGA patient data set. *MMP9*, *CD44*, *DLL4*, *FOXP1*, *MERTK*, and *PTPRC* genes were found more expressed in tumors re-growing after cisplatinum treatment than in untreated tumors. We here describe a new *in vivo* ovarian carcinoma experimental setting that will be instrumental for specific trials of combination therapy to counteract cisplatinum resistance in order to improve the prognosis of ovarian patients.

## INTRODUCTION

Epithelial ovarian cancer (EOC) is a serious medical problem, with more than 100,000 women dying per year in western countries [1]. The relatively asymptomatic nature of ovarian cancer and the lack of adequate screening tests result in 75% of patients being diagnosed at late FIGO stages (III and IV). Standard treatment involves cytoreductive surgery followed by chemotherapy. Taxol and platinum compounds are the standard adjuvant therapy in EOC and have greatly improved overall survival (OS) with 70% of patients achieving complete remission after first-line platinum-based therapy. Unfortunately, almost all patients relapse with a resistant disease. Tumors are clinically classified as responsive or not to a platinum (DDP)-based therapy depending on the time to relapse from the end of adjuvant therapy. Specifically, they are classified as refractory if progressing during treatment, resistant if relapsing within twelve months, and sensitive if relapsing after twelve months [2]. This classification is important as it dictates the subsequent chemotherapy. Understanding the molecular mechanisms of the sensitivity or resistance to DDP will help in tailoring ovarian cancer treatment, and possibly to improve the prognosis. The molecular mechanisms of resistance to a DDP-based therapy are multifactorial, and include mechanisms interfering with drug transport, with the repair of the DDP-induced DNA damage, with DDP-induced signalling to the apoptotic machinery, up- and down-regulated expression of miRNAs, and others [3]. Both the activation of the epithelial-mesenchymal transition (EMT) pathway and the existence of cancer stem cells (CSCs) have been advocated as possible mechanisms of relapse in different tumor types, including ovarian carcinomas [4–5]. However, most of these data come from cell cultures assays, and few studies are based on paired primary and recurrent epithelial ovarian cancer samples [4–10]. More appropriate *in vivo* models are needed to recapitulate the primary and secondary/acquired DDP resistance in ovarian cancer patients.

We recently characterized a panel of patient-derived xenografts (PDXs) from fresh ovarian tumor samples transplanted in nude mice [11]. These PDXs well reproduce the biological behaviour of the disease, including the heterogeneous response to a platinum-based therapy. Here we describe an experimental setting in which ovarian PDX-bearing mice were treated with one cycle of cisplatinum (cDDP), consisting of the drug given once a week for three weeks. Then, the regrowing tumors were re-challenged with a second cycle of treatment. These experiments clearly demonstrate not only that cDDP has a wide range of efficacy, as already reported, but that tumors regrowing after one cDDP treatment are less sensitive to a second cycle. In this experimental setting, we investigated the role of genes involved in EMT and stemness pathways in the response to cDDP.

## RESULTS

### Response of serous/endometrioid ovarian carcinoma xenografts to cDDP

**Figure 1:**
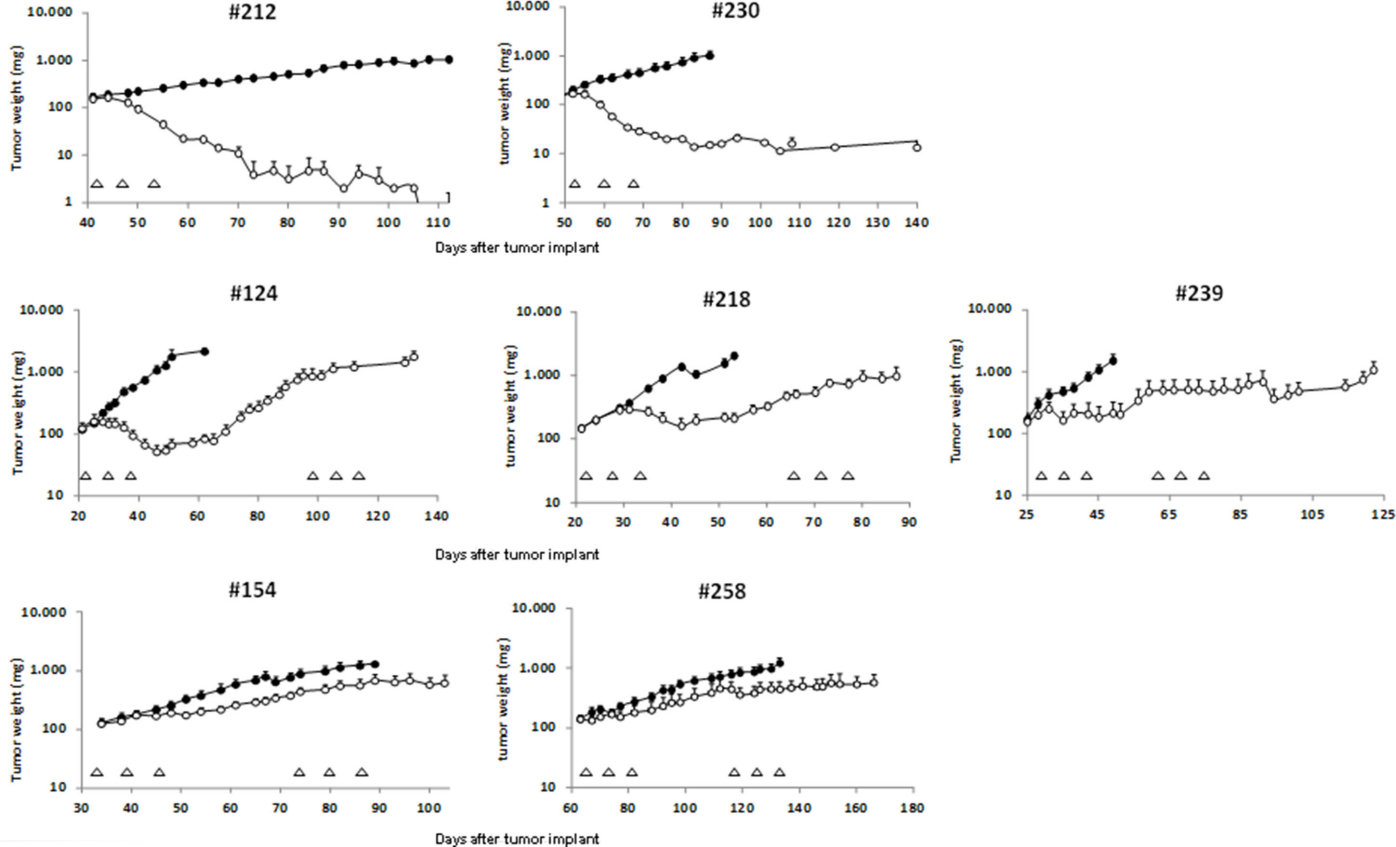
Tumor growth inhibition after cDDP treatment in ovarian PDXs The different xenografts were subcutaneously transplanted in nude mice and when tumor masses reached the weight of 120 mg, mice were randomized to receive vehicle or cDDP as specified on Materials and Methods. Graphs represent the tumor growth curves of each ovarian PDX treated (-◦-) or not (-●-) with cDDP. Data are the mean ± standard error of the tumor weight (mg) of each experimental group at different time points after tumor transplant. Each triangle indicates a cDDP treatment (one cycle consisting of three weekly treatment), and each group consisted of 8–10 mice.

We selected seven high grade serous/endometrioid ovarian PDXs (Table 1) of our recently established ovarian xenobank [11]. The characteristics of the patients from whom the xenografts were derived are summarized in Supplementary Table 1. We focused on these two high grade tumor histotypes as they represent the majority of ovarian carcinomas, and have similar clinical courses and responses to therapy. Of note, the two endometrioid PDXs were obtained from relapsing patients, likely with a more aggressive phenotype. These xenografts were challenged for the response to cDDP. A first cycle of cDDP was given by intravenous injection once a week for three weeks at the dose of 5 mg/kg. As depicted in Table 1 and Figure 1 different responses to cDDP treatment were observed. Xenografts #212 and #230 were extremely sensitive to cDDP treatment (T/C% values of 0.9% and 1.2%, respectively), showing not only tumor regressions, but also cures with 6 out of 9, and 6 out of 8 mice cured, respectively. These PDXs were classified as Very Responsive (VR) (Table 1). Xenografts #124, #218 and #239 were classified as Responsive (R) to cDDP with T/C% values of 3.8%, 10.3%, and 14.2%. In particular, these tumors underwent regressions after the first cycle of cDDP, but eventually they all re-start to grow. Xenografts #154 and #258 were less responsive to cDDP, with T/C% of 38.5% and 36.5%, with no sign of regression following cDDP treatment. They were classified as Low Responsive (LR). The different responses to cDDP did not seem to be dependent on tumor growth, as VR and LR xenografts had similar growth rates (Supplementary Figure 1).

**Table 1:** cDDP activity in ovarian PDXs

		cDDP Response	
ID	Histotype	T/C% (day)	Xenograft classification
#212	serous/endometrioid	0.9 (108)	**VR**
#230	endometrioid	1.2 (105)	**VR**
#124	serous/endometrioid	3.8 (51)	**R**
#218	endometrioid	10.3 (53)	**R**
#239	serous	14.2 (49)	**R**
#154	endometrioid	38.5 (67)	**LR**
#258	serous	36.5 (133)	**LR**

VR: Very Responsive; R: Responsive; LR: Low Responsive.

**Figure 2:**
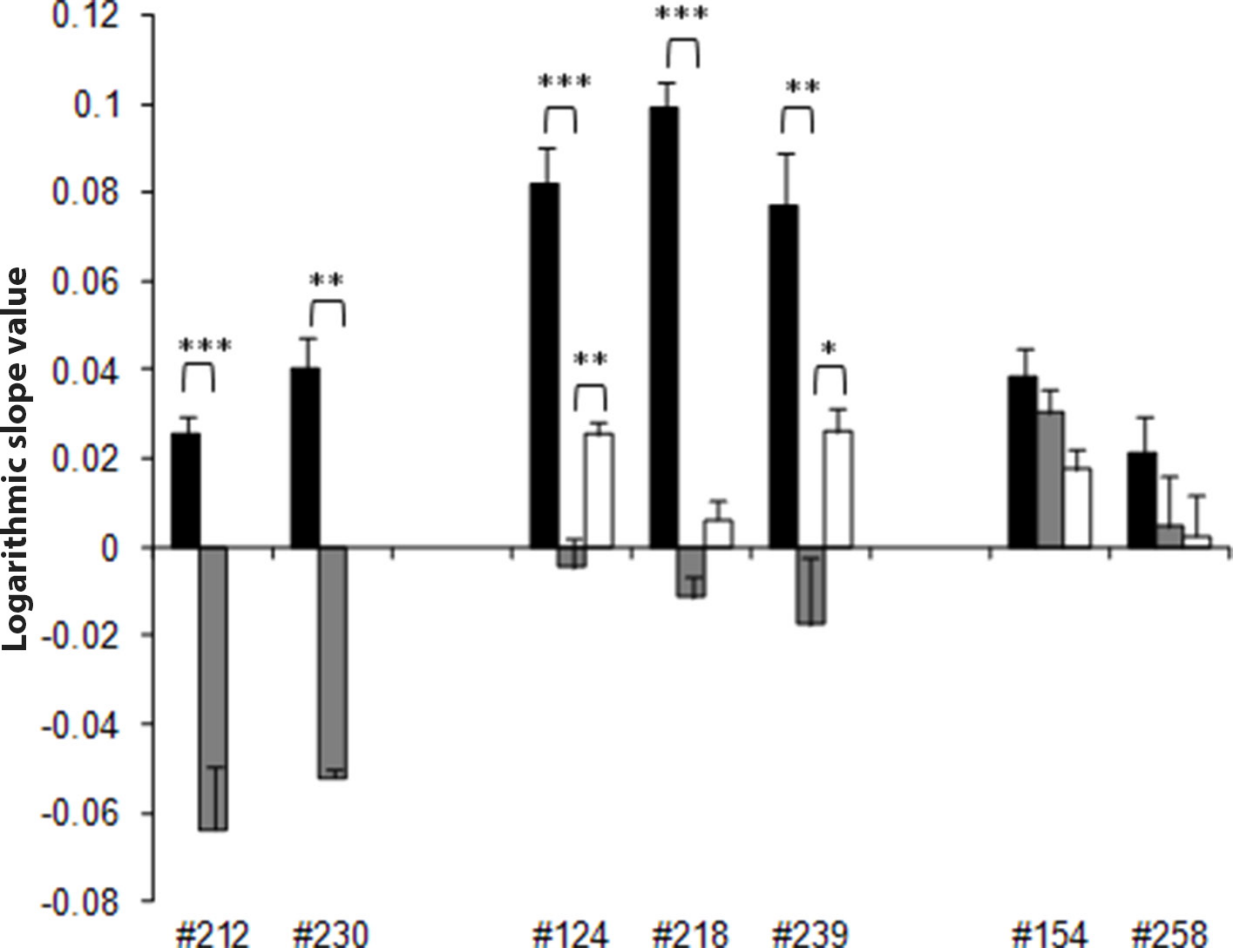
Quantification of cDDP antitumor effect in the different ovarian cancer PDXs The calculation of T/C% to quantify the effect of the second cycle of cDDP was not possible, as untreated control mice were already sacrificed, and we calculated the slopes of the interpolation line in all the different experimental groups as specified in Materials and Methods. The histograms represent the mean ± standard error of the slope of the interpolation lines in untreated/control and cDDP-treated groups (■-CTR, ■-1st cDDP cycle, and □-2nd cDDP cycle) in ovarian cancer xenografts (MNHOC212, MNHOC230, MNHOC124, MNHOC218, MNHOC239, MNHOC154 and MNHOC258). *p < 0.05, **p < 0.005, ***p < 0.0005.

When a second cycle of cDDP was given to the tumors regrowing after the first cycle, the antitumor activity was lower, especially in R xenografts. In order to evaluate the response to the second cDDP treatment, the slopes of the interpolation lines of the tumor growth curves were considered. Indeed, the calculation of the T/C% was not possible as most of the mice belonging to the control group was already dead by the time of the second cDDP cycle. Figure 2 reports the values of the different slopes obtained in untreated and cDDP-treated tumors (after the first and the second cycle). In particular, after the first cycle VR and R tumors (#212, #230, #124, #218, and #239) showed regressions, as suggested by the negative values of tumor growth slopes. On the other hand, positive slopes were observed in R xenografts #124, #218, and #239 after the second cDDP cycle, indicating a lower drug response. In LR xenografts (#154, #258) slopes were no statistically different in control-untreated and treated with one or two cDDP cycles.

### EMT and CSCs gene expression and response to cDDP

The experimental setting described above, and the different cDDP responses observed in the PDXs prompted us to investigate the role of EMT and CSCs-related genes in the responses to cDDP. The expression of these genes was investigated using high-throughput 384-well plates pre-filled with primers for EMT/CSCs genes in untreated and relapsing cDDP treated-tumors of VR (#212, #230), R (#124, #218, #239) and LR (#154, #258) xenografts. This approach has already been used successfully [12, 13]. All the regrowing-treated tumors were collected after a mean of 33 days after the last cDDP treatment (range 14–45 days), so any short-term effect of drug treatment can be reasonably excluded.

**Figure 3:**
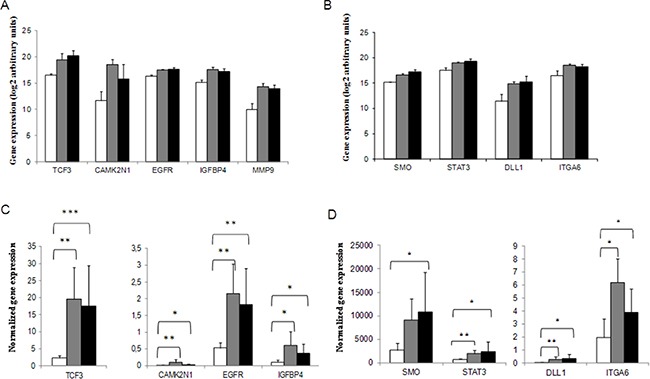
EMT and CSCs related genes differentially expressed in Very Responsive, Responsive and Low Responsive PDXs EMT (panel **A**) and CSC-related genes (panel **B**) found to be predictive of cDDP response. Real-time PCR data were pre-processed using the geometric mean of the available and appropriate housekeeping genes as endogenous control (*ACTB*, *B2M* and *HPRT1* for EMT plates; *ACTB*, *B2M*, *GAPDH*, *HPRT1* and *RPLP0* for CSCs plates). Data were expressed as arbitrary base-2 logarithmic units (2-deltaCt) in Very Responsive (□, VR), Responsive (■, R) and Low Responsive (■, LR) xenografts. EMT (panel **C**) and CSCs genes (panel **D**) validations by real-time PCR using custom-designed primers. Data are expressed as gene expression values normalized by the expression of housekeeping genes as specified in Materials and Methods. *p < 0.05, **p < 0.005, ***p < 0.0005.

To assess the predictive role of genes involved in these pathways, the LR tumor group was enriched with samples from cDDP-treated relapsing xenografts, as they all were less sensitive to the drug (Figure 2). Five EMT related genes were expressed at higher levels in LR xenografts (Figure 3, panel A). In particular, *TCF3* (Transcription Factor 3, *p* = 0.006) showed an increasing gene expression from VR to LR xenografts, while *CAMK2N1* (Calcium/calmodulin-Dependent Protein Kinase II Inhibitor 1, *p* = 0.03), *EGFR* (Epithelial Growth Factor, *p* = 0.0004), *IGFBP4* (Insulin-like Growth Factor Binding Protein 4, *p* = 0.002) and *MMP9* (Matrix-Metalloproteinase 9, *p* = 0.00013) had a lower expression in VR than R and LR tumors. The analysis on CSCs genes showed that 17 genes were differently expressed in LR, R and VR xenografts (data not shown). The genes differentially expressed among VR, R and LR that present the lowest *p* values were the following: *SMO* (Smoothened-Frizzled class receptor, *p* = 8.41E-05), *STAT3* (Signal Transducer and Activator of Transcription 3, *p* = 0.0011), DLL1 (Delta-Like 1, *p* = 0.0035), and *ITGA6* (Integrin, alpha 6, *p* = 0.0037) (Figure 3, panel B). The expression of these genes was higher in R/LR than in VR. The increased expression of all these genes, except *MMP9* (*p* = 0.2526) was validated using *ad hoc*-designed primers (Figure 3, panels C and D).

### Prognostic value of genes found to be predictive of cDDP response in ovarian cancer patients

Having found that some genes involved in EMT and CSCs pathways were predictive of cDDP response in our experimental system, we looked for correlations between their expression and survival in patients. Data of genome-wide gene expression and survival of high grade ovarian cancer patients were downloaded from the TCGA database. In particular, 261 patients were available for survival analysis. After a median follow-up of 57.6 months (IQR: 21.2–115.9 months), 147 (56.3%) deaths were registered. As reported in Table 2 and showed in Supplementary Figure 2, STAT3 was strongly associated with OS (HR = 13.68; *p* = 0.013), with higher levels being associated with a lower survival. In addition, the analysis demonstrated that the HR was dependent on time (time interaction *p* = 0.014). Specifically, the increase of the expression of *STAT3* significantly correlated with an increased HR up to the 12 months considered (χ^2^ = 10.049, *p* = 0.002 for six months, and χ^2^ = 9.797, *p* = 0.002 up to 12 months; Supplementary Table 2). This association was lost when considering the maximum observation period (60 months; χ^2^ = 1.254, *p* = 0.263; Supplementary Table 2).

**Table 2:** Correlation between survival and gene expression in patients from the TCGA dataset

Gene	Predictor variable	Term of the Cox model	HR	95% CI		*p*-value
**STAT3**	**gene**	**Linear**	**13.68**	**1.754**	**106.790**	**0.013**
	gene*log10(time)	**Interaction**	**0.41**	**0.203**	**0.837**	**0.014**
IGFBP4	gene	Linear	1.16	0.98	1.38	0.085
	*Note: interaction p-value = 0.418*					
TCF3	gene	Linear	1.05	0.82	1.35	0.692
	*Note: interaction p-value = 0.466*					
SMO	gene	Linear	1.07	0.91	1.26	0.424
	*Note: interaction p-value = 0.511*					
EGFR	gene	Linear	1.04	0.90	1.21	0.580
	*Note: interaction p-value = 0.082*					
ITGA6	gene	Linear	0.86	0.71	1.05	0.137
	*Note: interaction p-value = 0.990*					
DLL1	gene	Linear	0.99	0.90	1.10	0.919
	*Note: interaction p-value = 0.967*					

HR: Hazard Ratio; CI: Confidence Interval.

### Expression of EMT and CSCs genes in cDDP-treated/regrowing versus untreated tumors

**Figure 4:**
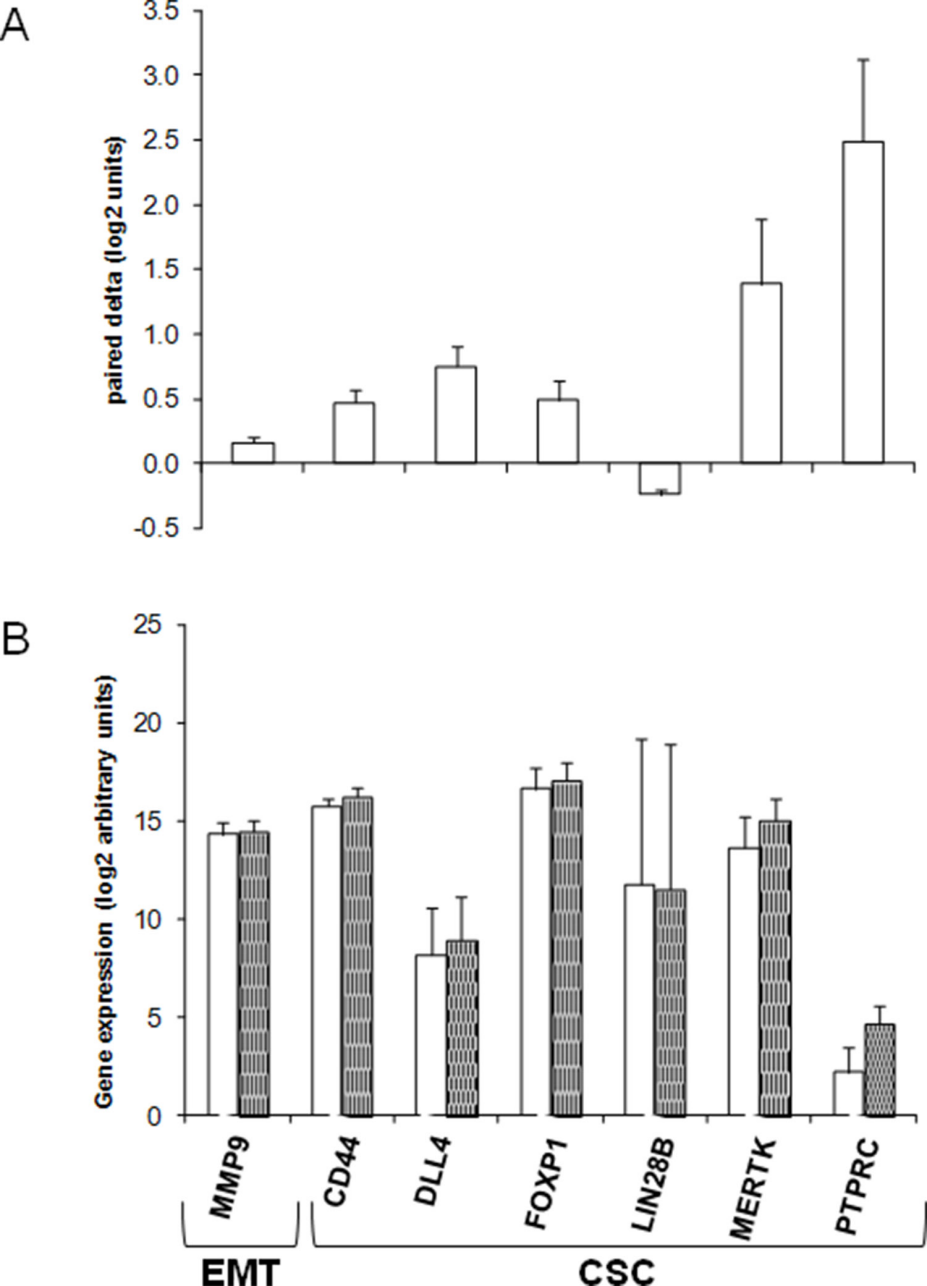
Differently expressed EMT and CSCs genes in cDDP-treated regrowing and control untreated Responsive ovarian cancer PDXs mRNA was extracted from control and treated tumor xenografts as described in Materials and Methods. (Panel **A**). Mean ± standard deviation of delta values (log_2_ units) of gene expression in paired cDDP-treated versus control/untreated xenografts. (Panel **B**). Mean ± standard deviation of the expression (log_2_ arbitrary units) in control (CTR, □) and in cDDP-treated (cDDP, ■) tumor xenografts of the genes found to be differentially expressed (paired *t*-test, *p* < 0.05) between the two experimental groups. Each group consisted of at least six replicates. All the real-time PCR data were pre-processed using the geometric mean of the available and appropriate housekeeping genes as endogenous control (*ACTB*, *B2M* and *HPRT1* for EMT plates; *ACTB*, *B2M*, *GAPDH*, *HPRT1* and *RPLP0* for CSC plates). Data were expressed as arbitrary base-2 logarithmic units (2-deltaCt).

The expression of EMT and CSCs genes was investigated in cDDP-treated regrowing and in the corresponding untreated xenografts in a comparative pair-wise analysis. The analysis was done only in R xenografts (#124, #218, and #239), as all these models were sensitive to the first cDDP challenge, and after regrowing were much less sensitive to the second one (Figure 2). Figure 4 shows the levels of the differently expressed EMT (*MMP9*) and CSCs genes (*CD44* antigen; *DLL4*- Delta like 4; *FOXP1*- Forkhead box P1; *MERTK*-C mer protocogene tyrosine kinase; *PTPRC*- Protein tyrosine phosphatase receptor type C, and *LIN28B*- Lin28 homolog B) (*p* < 0.05) and the corresponding relative delta values (log_2_). All the genes, except *LIN28B* that showed a downregulation, were upregulated in the cDDP-treated relapsing relative to untreated xenografts.

## DISCUSSION

Epithelial ovarian cancer is highly responsive to a DDP-based front-line therapy, but in most cases patients relapse with a resistant disease. This work describes a new experimental *in vivo* setting of high grade serous/endometrioid ovarian PDXs to study the molecular determinants of platinum sensitivity and the development of its resistance. The data herein reported can be summarized as follows: 1) the high grade serous/endometrioid ovarian carcinoma xenografts showed different sensitivity to cDDP, allowing their classification as VR, R and LR; interestingly enough, R xenografts regrowing after one cDDP cycle were much less responsive to a second treatment. Both the different cDDP responses of PDXs and the fact that cDDP-treated regrowing tumors are less responsive to a second cDDP cycle mirror the clinical setting; 2) among the EMT and CSCs-related genes found to be associated to the response to cDDP, *STAT3* could be validated in a cohort of ovarian patients; 3) in cDDP-treated regrowing tumors the levels of several genes involved in EMT and stemness were increased; 4) these results were obtained in both high grade serous and endometrioid histotypes suggesting a possible role of EMT and CSCs also in this latter type of tumor.

This experimental setting relies on PDXs recently stabilized in our laboratory [11], and shows how the cDDP activity can be monitored in a longitudinal way within the same cohort of mice bearing ovarian PDXs for a long time (up to 150 days). These models were exploited to better understand the molecular mechanisms of cDDP resistance. Indeed, cDDP responsive PDXs are much less sensitive to a second cDDP cycle, mirroring the clinical setting [2], reinforcing the value of these PDXs for *in vivo* studies on cDDP resistance. Most data on DDP resistance are based on *in vitro* cell lines, and very few *in vivo* results exist on gene modulation in representative ovarian xenografts models [14–16]. Considering that both the EMT and stemness pathways have been variably associated with DDP-resistant relapses in ovarian carcinomas [17, 18], we wondered whether the genes involved in these pathways could help predicting the platinum response in our experimental setting. Unlike other studies whose observation time was short [15], we focused on the expression of these genes in tumors regrowing after two cDDP cycles, a situation that mirrors tumor ovarian relapses in the clinic much better.

Four EMT related genes, *TCF3*, *CAMK2N1*, *EGFR*, and *IGFBP4*, were significantly more expressed in LR than in R and VR xenografts. The greater expression of *TCF3*(E2A) and *CAMK2N1* might underlie the activation of the Wnt pathway by both the canonical/β-catenin dependent way and the non-canonical one [19]. The over-expression of *EGFR* was reported to be involved in EMT activation via the Akt/Erk1/2 pathways, inducing Vimentin expression and dowregulation of E-cadherin favoring invasiveness in ovarian cancer cells [20]. On the other hand, the recently suggested role of EGFR in the repair of DNA damage induced by IR and cDDP suggests an increased repair in LR xenografts [21]. IGFBP4 is an important senescence-inducing factor in mesenchymal-stem cells (MCS), reducing cell growth and is associated with a lower DNA-damage response and less nuclear phospho/activated-ERK in senescent than young MSCs [22]. IGFBPs, by recruiting IFG proteins, inhibit their ability to activate their receptor IGFR, and downstream regulators such as PI3K and ERK [23]. We might speculate that the overexpression of *IGFBP4* in R and LR xenografts by inducing senescence or breaking the cell cycle in tumor cells might enable them to counteract cDDP activity.

Among the genes involved in stemness whose upregulation inversely correlated with the cDDP response in the xenografts tested, we found those engaged in Hedgehog (i.e. *SMO*), and Notch (*DLL1*) pathways. These pathways are also involved in the regulation of the EMT and are reported to be associated with drug resistance in different tumors, including ovarian tumors [24, 25]. Selective inhibition or knockdown of *SMO* and other Hedgehog downstream effectors (e.g. Gli1) increased cDDP sensitivity in ovarian cancer cell cultures and xenografts [26].

The role of STAT3 in cancer development and drug response has been recently reviewed [27]. Its inhibition has been shown to have antitumor activity, and to reverse the sensitivity to therapy in different cancer types [28–30], including ovarian cancer [31, 32]. Recently, a STAT3 role in DDP resistance and acquisition of stem-like features by STAT3 activation has also been described [33]. We found that *STAT3* expression was inversely correlated with cDDP response, and that its low levels predicted an increased overall survival in DDP treated ovarian cancer patients up to 12 months from diagnosis. The predictive value of STAT3 was lost over time, but patients with high STAT3 tumor level might have an higher risk of early relapse and could be treated more extensively.

The role of Notch signaling in the development of platinum and paclitaxel resistance has been reported in ovarian cancer [34, 35]. Despite the negative results of a phase II study of RO4929097, a gamma-secretase inhibitor, as single agent in recurrent-platinum resistant ovarian cancer [36], further studies are warranted to test Notch inhibitors in combination with chemotherapy. The increased expression of *ITGA6* we found predictive of cDDP response is in line with the literature as well. *ITGA6* expression levels were indeed associated with the presence of cancer stem-like cells [37, 38], as well as with an invasive phenotype, with drug resistance and poor prognosis [39] in a metastatic xenograft model [40]. Scant data exist on its role in predicting DDP response in ovarian cancer [41].

When we looked for genes upregulated in regrowing than “primary” tumors in a comparative pair-wise analysis, we found genes belonging to the extracellular matrix components (i.e. *MMP9*), to CSCs self-renewal maintenance (i.e. *FOXP1*), CSCs markers (i.e. *CD44*, *PTPRC*), and gene involved in MER-TK signaling pathway; the only downregulated gene was *LIN28B*. *MMP9* mRNA expression was found enhanced in the ascites of chemoresistant ovarian cancer patients [42], and a recent meta-analysis indicated its positive association with poor prognosis in ovarian cancer [43]. *FOXP1* expression was an independent risk factor associated with chemotherapy resistance and the prognosis of patients with ovarian cancer [44]. Among the CSC-related genes, to our knowledge no data are available on the role of *MERTK* in ovarian cancer. However, its over-expression has been associated with tumor progression and therapy response in lung cancer and leukemia [45–47]. We detected increased *CD44* expression in treated regrowing xenografts, corroborating its recently reported higher expression in recurrent and metastatic ovarian samples than their primary counterparts [48]. However, even CD44+ cell enrichment has been reported in the majority of ovarian cancer samples after neoadjuvant therapy, no significant association with chemoresistance was reported and a decrease in CD44 expression was associated with shorter survival [49].

The present findings corroborate the utility of ovarian PDXs for studying the molecular determinants of cDDP response. In this experimental setting, mice bearing ovarian PDXs were treated with different rounds of cDDP, and followed in a longitudinal way, mirroring the clinical situation. We found that the expression of some genes is associated with the response to cDDP in both high grade serous and endometrioid tumors, opening the way to test specific target inhibitors to increase cDDP activity in these tumor histotypes. The enrichment of genes involved in the EMT and CSC pathways underscore their importance in cDDP-treated re-growing tumors, although the specific molecular mechanisms at their basis (selection or induction) have still to be defined. The PDXs models we have set up will be instrumental for addressing specific trials of combination therapy to counteract resistance to cDDP based therapy with the final aim of improving the prognosis of ovarian cancer patients.

## MATERIALS AND METHODS

### Animals

Female NCr-nu/nu mice obtained from ENVIGO RMS srl (Correzzana, Italy) were used when 6–8 weeks old. They were maintained under specific pathogen-free conditions, housed in isolated vented cages, and handled using aseptic procedures. The IRCCS-Istituto di Ricerche Farmacologiche Mario Negri adheres to the principles set out in the following laws, regulations, and policies governing the care and use of laboratory animals: Italian Governing Law (D. lg 26/2014; Authorization n.19/2008-A issued March 6, 2008 by Ministry of Health); Mario Negri Institutional Regulations and Policies providing internal authorization for persons conducting animal experiments (Quality Management System Certificate-UNI EN ISO 9001:2008 – Reg, No.6121); the NIH Guide for the Care and Use of Laboratory Animals (2011 edition) and EU directives and guidelines (EEC Council Direcrive 2010/63/UE). The Statement of Compliance (Assurance) with the Public Health Service (PHS) Policy on Human Care and Use of Laboratory Animals was recently reviewed (9/9/2014) and will expire on September 30, 2019 (Animal Welfare Assurance #A5023-01).

### Xenograft models

We have recently characterized and stabilized a xenobank of ovarian carcinoma from fresh tumor samples. As reported, these PDX models recapitulate the tumor from which they derived and maintained these characteristics for multiple passages [11]. From this xenobank we selected seven PDXs: MNHOC124, MNHOC154, MNHOC218, MNHOC212, MNHOC230, MNHOC239, and MNHOC258 (referred to as #124, #154, #218, #212, #230, #239, and #258), whose main characteristics are reported in Supplementary Table 1. Viable tumor fragments (2 × 2 mm) were subcutaneously (s.c.) implanted through trocar needles and mice were randomized when the average tumor size was about 120 mg (8–10 per group, on the basis of pragmatic considerations). Cisplatinum (cDDP, Sigma-Aldrich) was given i.v. at the dose of 5 mg/kg once a week for three times (q7d × 3). The second cDDP cycle treatment was generally given when tumors started to re-growth (when tumor weights were ≥ 3 fold those at randomization), and were in the range of 400–1000 mg. Tumor growth was measured twice a week with a Vernier caliper, and tumor weights (mg = mm^3^) were calculated as follows: (length [mm] * width [mm]^2^)/2. The efficacy of the treatment was expressed as best tumor growth inhibition [%T/C = (tumor weight mean of treated tumors/tumor weight mean of control tumors) * 100]. Drug activity was defined as follows: low responsive (LR) with T/C% ≥ 30% and no regression, responsive (R) with 10% < T/C% < 30% with observed regressions followed by tumor re-growth, and very responsive (VR) with T/C% ≤ 10% and with regressions but no tumor re-growth.

The cDDP effect was also quantified extrapolating the slope of the growth curves of the control/untreated and cDDP-treated tumors. The tumor growth curves for each mouse were plotted on a logarithmic scale, and the slope of the interpolation line was calculated; the mean ± standard error of the slopes for each group (control, first cycle and second cycle-treated; these latter slopes were calculated considering the re-growing phase of the tumours after the first cDDP cycle treatment) was calculated and plotted as histograms. Statistical analysis was done by one-way ANOVA test, with GraphPad Prism 3.01 software.

### High-throughput gene expression real time assay

Total mRNA was extracted from snap-frozen tissues by using Maxwell 16 LEV SimplyRNA (Promega), according to manufacturer protocols. Control/no treated (CTR) and cDDP-treated re-growing tumors (after the second cDDP cycle) were snap-frozen at the time of sacrifice, when tumor weights were about 400–1000 mg. The RT^2^ Profiler PCR Arrays (Qiagen) are designed to analyze a panel of genes related to EMT and CSCs. Each array contains a panel of 4×96 primer sets for a thoroughly researched set of 84 genes, plus five housekeeping genes, three mRNA retrotranscription and three PCR quality controls. For each plate two control/no-treated and two cDDP-treated samples of the same xenograft were included, except for MNHOC212 and MNHOC230 for which only control no-treated samples were analyzed. The assay includes a specific step of mRNA retro-transcription, using the RT^2^ First Strand Kit. The plate was filled by an EP Motion 5075 robot (Eppendorf), so an excess of volume was used. Reactions were done on a 7900HT Sequence Detection System (Applied Biosystems).

### Data analysis and validation

Real-time PCR data were pre-processed with DataAssist software (v.2, Life Technologies), using the geometric mean of the available and appropriate housekeeping genes as endogenous control (*ACTB, B2M* and *HPRT1* for EMT plates; *ACTB, B2M, GAPDH, HPRT1* and *RPLP0* for CSC plates). Data were expressed as arbitrary base-2 logarithmic units (2-deltaCt). Statistical analysis was done with the tMEV suite (http://www.tm4.org [50]) using one-way ANOVA for the genes associated with responsiveness, and a paired *t*-test for the long-term effects of cDDP on the responsive xenografts. Validation assays were done by real-time PCR with *ad-hoc* designed primers (Primer3, http://primer3.ut.ee/) only for the analysis of the predictive value of genes, as the different samples run on different plates. Gene expression data were quantified through a calibration curve, and were normalized by gene expression of an housekeeping gene (HPRT1). Statistical analysis was done by *t*-test, using GraphPad Prism 3.01 software.

### Survival analysis

Gene expression (IlluminaHISeq UNC platform) and survival data were collected from The Cancer Genome Atlas (TCGA,http://cancergenome.nih.gov/) in May 2015. Overall Survival (OS) was defined as the time from diagnosis to the time of death from any cause; data was right-censored if a patient was alive at the last follow-up. The hazard ratio (HR) was used as summary statistics correlating gene expression with OS. A linear log_10_ time-by-gene interaction term was introduced into the Cox regression model to detect a time varying HR. In case of curvature over time of the relative hazard function, the restricted mean to time *t* * was used as summary statistic correlating gene expression with OS; *t* * was considered as 6 months (patients relapsing by 6 months are classified as resistant to therapy), 12 months (patients relapsing between 6 and 12 months are classified as responsive to therapy), and 60 months (slightly below the maximum collected follow-up time). Tertiles of gene expression levels were used to plot and describe survival.

F. Ricci is a recipient of a fellowship from the Italian Foundation for Cancer Research (FIRC).

## SUPPLEMENTARY MATERIALS FIGURES AND TABLES


