# Supplementary material for: Patient-derived ovarian cancer xenografts re-growing after a cisplatinum treatment are less responsive to a second drug re-challenge: a new experimental setting to study response to therapy
Source: Oncotarget. 2016 Feb 17;8(5):7441–51. doi: 10.18632/oncotarget.7465 (PMC5352333; doi:10.18632/oncotarget.7465)
Supplement: Supplementary file 1 [file oncotarget-08-7441-s001.pdf]

Patient-derived ovarian cancer xenografts re-growing after a cisplatinum treatment are less responsive to a second drug re-challenge: a new experimental setting to study response to therapy

Supplementary Materials

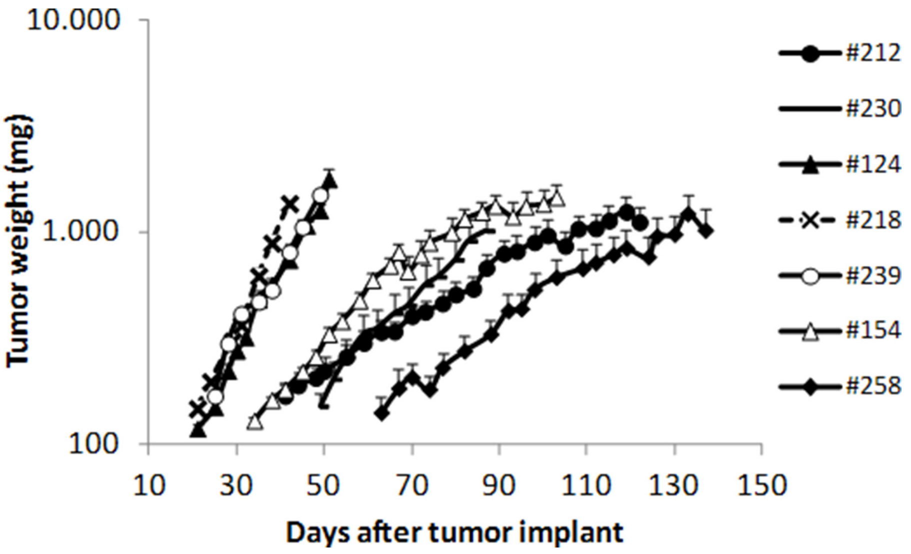

Supplementary Figure S1: Tumor growth curves of PDXs under study. Tumor growth curves expressed as tumor weight (mg) versus time (days after tumor implant) for the seven xenografts subcutaneously injected in nude mice.

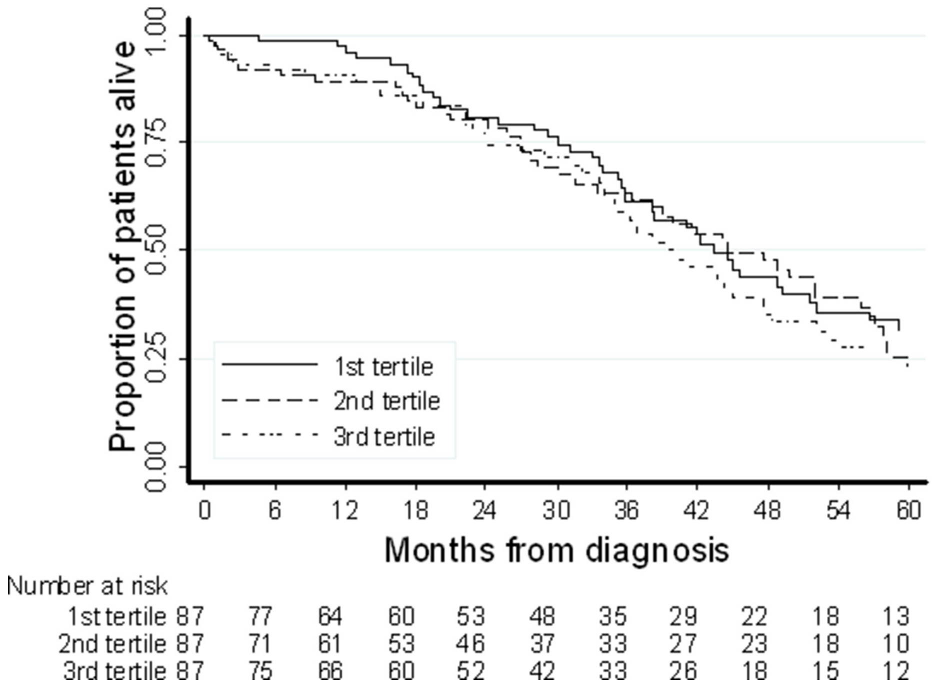

Supplementary Figure S2: *STAT3* gene expression and OS in 261 patients of the TCGA dataset. Tertiles of the gene expression of *STAT3* were used to plot survival.

Supplementary Table S1: Characteristics of the patients from whom the PDXs originally derived

XENO ID	AGE (years)	HISTOTYPE	GRADE	STAGE	°ORIGIN	TREATMENT	RESPONSE TO CHEMOTHERAPY$	SURVIVAL (months)
**#124**	73	serous/ endometrioid	G2	IIIC	P	CBDCA/PTX	N	9
**#154**	42	endometrioid	G2	IIC	R	CBDCA/PTX	Y	101
**#212**	55	serous/ endometrioid	G2	IIIC	P	CBDCA/PTX	Y	23
**#218**	39	endometrioid	G3	IIIC	P	CBDCA/PTX	Y	18
**#230**	50	endometrioid	G3	IIB	R	CBDCA/PTX	Y	40
**#239**	71	serous	G2	IV	R	CBDCA/PTX	Y	40
**#258***	74	serous	G3	IIIC	P	-	-	-

*the patient died soon after surgery due to septic shock;

°P: primary; R: relapse;

$Response to first line therapy (adjuvant neoadjuvant therapy); Y: sensitive tumor (relapsing after 12 months); N: resistant tumors (relapsing in 0–6 months).

Supplementary Table S2: Survival analysis on genes correlated to overall survival from the TCGA database

Gene	Statistics	Category	Point estimate	95%CI	χ^2	*p*-value^
STAT3	RM°–6 months	10.4–12.0	6.0	5.9–6.0	10.049	0.002
		12.0–12.5	5.7	5.4–5.9		
		12.5–13.6	5.7	5.4–5.9		
	RM°–12 months	10.4–12.0	11.9	11.7–12.0	9.797	0.002
		12.0–12.5	11.1	10.5–11.7		
		12.5–13.6	11.2	10.6–11.7		
	RM°–60 months	10.4–12.0	42.2	38.2–46.3	1.254	0.263
		12.0–12.5	40.7	36.2–45.3		
		12.5–13.6	38.9	34.6–43.1		

°RM: restricted mean to time *t**

^*p*-value for trend.
